# Antibiotics with Interleukin-15 Inhibition Reduce Joint Inflammation and Bone Erosions but Not Cartilage Destruction in Staphylococcus aureus-Induced Arthritis

**DOI:** 10.1128/IAI.00960-17

**Published:** 2018-04-23

**Authors:** Berglind Bergmann, Pernilla Jirholt, Petra Henning, Catharina Lindholm, Claes Ohlsson, Iain B. McInnes, Ulf H. Lerner, Inger Gjertsson

**Affiliations:** a Department of Rheumatology and Inflammation Research, Institute of Medicine, Sahlgrenska Academy at University of Gothenburg, Gothenburg, Sweden; b Centre for Bone and Arthritis Research, Department of Internal Medicine and Clinical Nutrition, Institute of Medicine, Sahlgrenska Academy at University of Gothenburg, Gothenburg, Sweden; c Glasgow Biomedical Research Centre, University of Glasgow, Glasgow, United Kingdom; Albert Einstein College of Medicine

**Keywords:** IL-15, S. aureus, arthritis, immunopathogenesis, osteoclasts, mice

## Abstract

Staphylococcus aureus-induced arthritis causes rapid joint destruction, often leading to disabling joint damage despite antibiotics. We have previously shown that interleukin-15 (IL-15) inhibition without antibiotics is beneficial in S. aureus-induced arthritis. We therefore hypothesized that the inhibition of IL-15, in combination with antibiotics, might represent a useful therapy that would reduce inflammation and joint destruction but preserve the host's ability to clear the infection. Female wild-type C57BL/6 mice were intravenously inoculated with the toxic shock syndrome toxin 1 (TSST-1)-producing LS-1 strain of S. aureus with 0.8 × 10^8^ CFU S. aureus LS-1/mouse. Three days later, treatment consisting of cloxacillin, followed by flucloxacillin, together with either anti-IL-15 antibodies (aIL-15ab) or control antibodies, was started. Studied outcomes included survival, weight change, bacterial clearance, and joint damage. The addition of aIL-15ab to antibiotics in S. aureus-induced arthritis reduced synovitis and bone erosions compared to controls. The number of bone-resorbing osteoclasts in the joints was reduced, whereas cartilage destruction was not significantly altered. Importantly, the combination therapy did not adversely affect the clinical outcome of S. aureus-induced arthritis, such as survival or weight change, or compromise the host's ability to clear the infection. Since the clinical outcome of S. aureus-induced arthritis was not affected, the addition of aIL-15ab to antibiotics ought to be safe. Taken together, the combination of aIL-15ab and antibiotics is a beneficial, but not optimal, treatment of S. aureus-induced arthritis since it reduces synovitis and bone erosions but has a limited effect on cartilage destruction.

## INTRODUCTION

Septic arthritis, most often caused by Staphylococcus aureus, is a medical emergency that leads to a rapid destruction of the bone and cartilage within the joint, culminating in irreversible joint damage if not promptly treated. Antibiotics and supportive care remain the mainstay of treatment. However, there is a need for therapy improvement since more than half of the treated patients develop permanent joint damage and dysfunction, despite eradication of the bacteria, due to the overwhelming host immune response to the pathogen (1, 2).

Synovial inflammation is closely related to articular bone destruction. Infiltrating leukocytes and resident cells within the joint produce mediators such as receptor activator of nuclear factor-κB ligand (RANKL), macrophage colony-stimulating factor (M-CSF), and proinflammatory cytokines such as interleukin-1 (IL-1) and tumor necrosis factor alpha (TNF-α), which leads to enhanced differentiation of bone-resorbing osteoclasts that mediate bone resorption (3). Osteoclast differentiation has been suggested to occur within the inflamed joint where osteoclast precursors of the monocyte lineage have migrated from the bone marrow into the secondary lymphoid organs and subsequently to the joints (4). The identity of the osteoclast precursor within the inflamed joint is unclear, but the Ly6C^high^ monocyte subset, sometimes referred to as inflammatory monocytes, is a candidate (5). Cartilage destruction in the inflamed joint is, on the other hand, mediated by the inflammation-triggered secretion of matrix-degrading enzymes such as metalloproteinases (6) and possibly also by the bacterium itself and its associated toxins (7).

IL-15 is a proinflammatory cytokine that has a wide variety of functions at the interface between innate and adaptive immunity and has been considered to play a role in the immunopathogenesis of many inflammatory, infectious, and autoimmune diseases (8). It has an essential role in the differentiation, proliferation, and activation of natural killer (NK) cells, as well as in the maintenance of peripheral memory CD8^+^ T cells (8), and is thus important during viral infections (9). IL-15 also enhances the phagocytic activity of macrophages and neutrophils and inhibits neutrophil apoptosis (10, 11). Furthermore, IL-15 is important for cell recruitment to inflamed areas through the induction of other proinflammatory cytokines and chemokines. For example, it stimulates human monocytes to produce monocyte chemoattractant protein 1 (MCP-1) and IL-8, chemokines that recruit monocytes and neutrophils, respectively, to the site of inflammation (11).

IL-15 has been implicated as a pro-osteoclastogenic cytokine (12–14) since it potentiates the *in vitro* differentiation of osteoclasts (12, 13). In addition, IL-15-activated NK cells from synovial fluid obtained from patients with rheumatoid arthritis could efficiently induce the formation of osteoclasts from monocytes (15). These NK cells expressed M-CSF and RANKL, which were further upregulated by IL-15. In patients with rheumatoid arthritis, IL-15 is detected at high concentrations in synovial fluid (16), and genetic variants of *IL-15* associate with progression of joint destruction in Caucasian (17) but not Japanese (18) patients. Thus, IL-15 may mediate bone resorption during inflammatory conditions.

Serum protein levels of IL-15 increase during experimental S. aureus-induced arthritis and sepsis (19), and we have previously shown that both IL-15 knockout mice and mice treated with a monotherapy of anti-IL-15 antibodies (aIL-15ab) developed a less destructive S. aureus-induced arthritis than control groups (19). Furthermore, in other mouse models of aseptic arthritis, as well as in Gram-negative and endotoxin-induced murine sepsis, inhibition of IL-15 has been beneficial with respect to both arthritis development (20, 21) and survival during sepsis (22, 23).

Since the host immune response, rather than the pathogen itself, is responsible for the residual joint dysfunction in S. aureus-induced arthritis, several attempts have been made to reduce the severity of the arthritis by targeting the immune response without adversely affecting host defense mechanisms. Antibiotics have been combined with corticosteroids without convincing effects on joint destruction in the mouse model of S. aureus-induced arthritis (24) but with beneficial effects in children with septic arthritis (25). Antibiotics have also been combined with biologics such as TNF-α inhibitors and IL-1 receptor (IL-1R) antagonist with limited success (26–28).

A delicate balance must be maintained between the immune system's ability to clear the infection and the risk of collateral damage mediated by an overwhelming immune response. We and others have previously shown that inhibition of IL-15 protects against sepsis (19, 22, 23), as well as against both septic and aseptic arthritis development (19–21). We therefore hypothesized that IL-15 might represent a beneficial treatment target to combine with antibiotics in the treatment of S. aureus-induced arthritis. The aim of the present study was to investigate whether the addition of anti-IL-15 antibodies to antibiotics could improve the outcome of S. aureus-induced arthritis compared to antibiotic treatment alone.

## RESULTS

### IL-15 inhibition added to antibiotic treatment does not adversely affect the systemic infection and systemic bone loss in S. aureus-induced arthritis.

**FIG 1:**
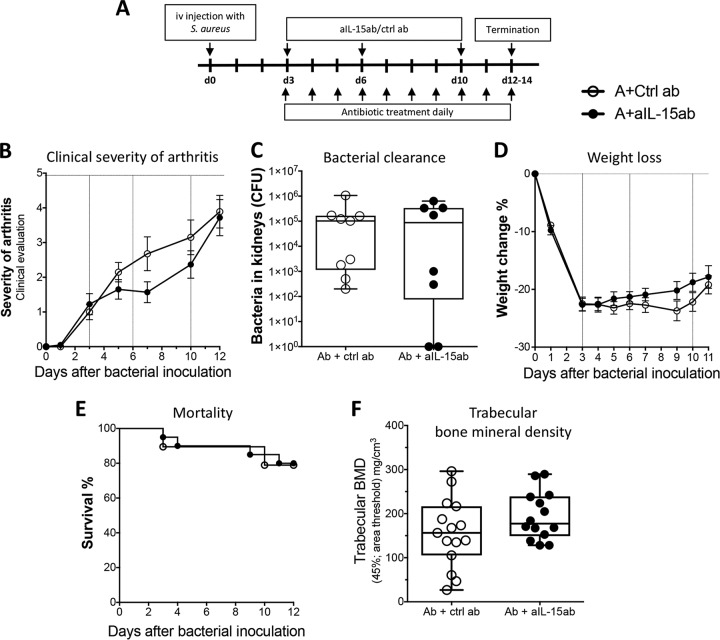
The addition of anti-IL-15 antibodies (aIL-15ab) to antibiotics does not adversely affect the clinical course of S. aureus-induced arthritis. (A) Mice were i.v. inoculated with S. aureus LS-1 and treated 3 days later with (i) aIL-15ab with antibiotics for the combination therapy group or (ii) control antibodies with antibiotics for the control group. The antibodies were injected intraperitoneally at days 3, 6, and 10 postinfection. (B) The severity of arthritis was evaluated during the course of the infection. (C) Bacterial persistence in the kidneys at 12 days postinfection. (D) Weight change as a percentage of initial weight. (E) Cumulative survival during the 12-day period. Data are representative of three separate experiments with 10 mice/treatment group. (F) Femur trabecular bone mineral density (BMD) in the metaphysical region was measured by pQCT. Values from two experiments were pooled, with 10 mice/treatment group. In panels B and D, bars show the means ± standard errors of the mean. In panels C and F, data are shown as medians; the whiskers represent minimum to maximum ranges. Statistical differences were calculated using a Mann-Whitney U test. Kaplan-Meier survival plots were prepared, and a log-rank test was used for comparison between the two survival curves. Abbreviations: A, antibiotics; ctrl ab, control antibodies; aIL-15ab, anti-IL-15 antibodies.

To evaluate the effect of a combination therapy of aIL-15ab with antibiotics on experimental S. aureus-induced arthritis, mice were intravenously (i.v.) inoculated with S. aureus, and 3 days later combination therapy consisting of antibiotics, together with either aIL-15ab or control antibodies, was started (Fig. 1A). At day 3 after bacterial inoculation, bone and cartilage destruction was already evident and progressed thereafter (29). The rationale for starting treatment at day 3 was to mimic the treatment delay which is present in the clinical setting of septic arthritis.

Inhibition of IL-15 combined with antibiotic treatment did not significantly affect the clinical severity (Fig. 1B) or frequency of arthritis (data not shown), bacterial clearance (Fig. 1C), weight loss (Fig. 1D), or mortality (Fig. 1E) during the infection compared to antibiotics with control antibodies. However, the monotherapy of aIL-15ab reduced bacterial clearance and, to a certain extent, also weight loss (19), but not at all to the same degree as when antibiotics were added (see Fig. S1A to D in the supplemental material). The results shown in Fig. S1C and D for groups without antibiotics have been previously published (19).

S. aureus-induced arthritis results in a prominent systemic bone resorption (30). To evaluate whether the combination therapy would influence this feature of the infection, peripheral quantitative computed tomography (pQCT) was performed on femoral bones at 12 days postinfection. Treatment with antibiotics and aIL-15ab did not significantly influence trabecular bone mineral density, cortical thickness, or density (Fig. 1F; see also Fig. S2A and B in the supplemental material). Thus, the addition of aIL-15ab to antibiotic therapy did not improve or adversely affect the clinical outcome of S. aureus-induced arthritis.

### Inhibition of IL-15 with antibiotic treatment reduces synovitis and bone erosions but not cartilage destruction in S. aureus-induced arthritis.

**FIG 2:**
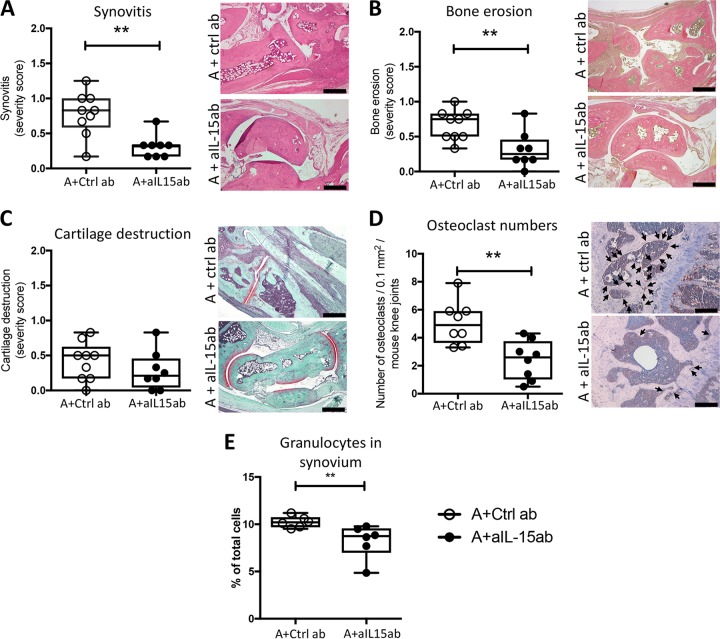
Antibiotics and IL-15 inhibition reduce synovitis, bone erosions, and osteoclast numbers in the joint but do not influence cartilage destruction during S. aureus-induced arthritis. (A to E) Effects of adding aIL-15ab to antibiotics on joint inflammation, bone erosions, and cartilage destruction, on the number of osteoclasts, and on the proportion of granulocytes in the synovium. Shown are representative stained sections (hematoxylin and eosin, Van Gieson, Safranin O, and cathepsin K) of inflamed joints on day 12 postinfection. The severity of joint inflammation, or synovitis, and joint destruction was evaluated by making a histological scoring of synovitis (A), bone erosions (B), and proteoglycan loss in the articular cartilage (C) and by counting the number of osteoclasts (arrows) in the epiphyseal part of femur and tibia after cathepsin K staining (D). (E) Proportion of granulocytes in the synovium. Data are representative of three separate experiments (*n* = 10 mice/treatment group). The horizontal bars in panels A to E show median values; the whiskers represent minimum to maximum ranges. Statistical differences were calculated using a Mann-Whitney U test. **, *P* < 0.01 compared to the control group. Scale bars, 100 μm. Abbreviations: A, antibiotics; ctrl ab, control antibodies; aIL-15ab, anti-IL-15 antibodies.

Since the clinical evaluation of arthritis has a limited sensitivity, we performed histopathological evaluation of joints at day 12 postinfection. Mice treated with the combination therapy of antibiotics and aIL-15ab developed less severe synovitis than did control mice treated with antibiotics and control antibodies, which was consistent with the finding of reduced proportions of granulocytes in the synovium (Fig. 2A and E). There were no differences between the two treatment groups in the proportions of lymphocytes, CD19^+^ B cells, T cells, NK cells, or NKT cells in the synovium (see Fig. S3 in the supplemental material). Importantly, mice that received the combination therapy of antibiotics and aIL-15ab had reduced bone erosions compared to control mice treated with antibiotics and control antibodies (Fig. 2B). There was no significant difference between the two groups with respect to cartilage destruction (Fig. 2C). Antibiotic treatment alone significantly reduced synovitis and bone destruction irrespective of aIL-15ab treatment; however, the effect was enhanced by the addition of aIL-15 ab (see Fig. S1E and F in the supplemental material). The results shown in Fig. S1E and F for groups without antibiotics have been previously published (19).

Osteoclasts are the only cells that resorb bone and are hence responsible for bone destruction in the inflamed joint. To evaluate whether the reduced bone erosion in the combination therapy group was due to an effect on osteoclast number, we performed cathepsin K staining of joint sections. Indeed, mice treated with antibiotics and aIL-15ab had fewer osteoclasts in their knee joints than controls (Fig. 2D).

To assess whether the combination therapy had an inhibitory effect on osteoclast numbers by interfering with the molecular triad of RANK, RANKL, and OPG (osteoprotegerin; a decoy receptor for RANKL), we measured their relative mRNA expression levels in synovia from the knee joints from day 12 postinfection. At this time point, no differences were observed between the groups (see Fig. S2C to E in the supplemental material).

Taken together, these observations indicate that the addition of aIL-15ab to antibiotic treatment during S. aureus-induced arthritis enhances the reduction of synovitis and bone erosions, but not the cartilage destruction. These findings coincide with a reduced number of granulocytes in the synovium and fewer osteoclasts in the joint.

### The combination therapy of aIL-15ab and antibiotics increases the proportion of inflammatory monocytes in the draining lymph nodes and decreases the serum levels of MCP-1.

**FIG 3:**
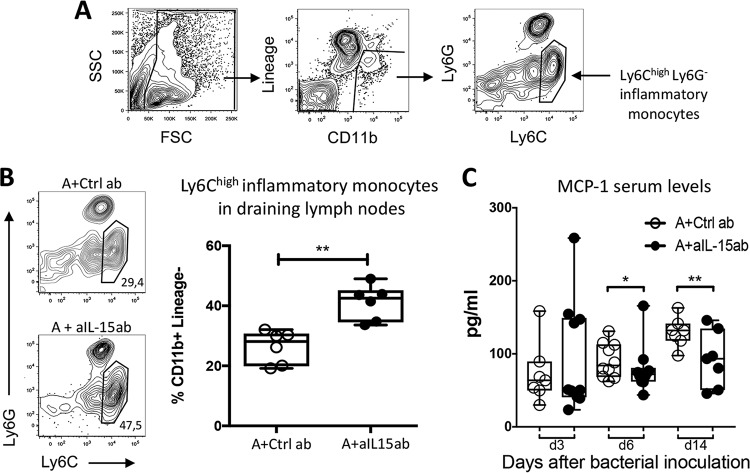
The combination therapy of aIL-15ab and antibiotics increases the proportion of inflammatory monocytes in the draining lymph nodes of the knee joints during S. aureus-induced arthritis. (A) Representative illustration of the flow cytometry gating strategy for the analysis of Ly6C^high^ inflammatory monocytes in the draining lymph nodes of the knee joints. Debris and doublets were excluded and cells were subgated on CD11b^+^ Lineage^−^ cells (gated out using a B220, CD4, CD8, and NK1.1 FITC dump channel). The remaining CD11b^+^ Lineage^−^ cells were then gated based on Ly6C and Ly6G expression, and inflammatory monocytes are defined as Ly6C^high^ and Ly6G^−^. (B) The proportion of inflammatory monocytes is higher in mice receiving aIL-15ab and antibiotics than in control mice 12 days after the initiation of S. aureus-induced arthritis. A representative flow cytometry analysis of the inflammatory monocyte subset in both treatment groups is shown. (C) Serum levels of MCP-1 at days 3, 6, and 14 after bacterial inoculation. Horizontal bars show median values; the whiskers represent minimum to maximum ranges. Statistical differences were calculated using a Mann-Whitney U test. *, *P* < 0.05 compared to the control group; **, *P* < 0.01 compared to the control group. Abbreviations: A, antibiotics; ctrl ab, control antibodies; aIL-15ab, anti-IL-15 antibodies.

The Ly6C^high^ inflammatory monocyte subset (Fig. 3A) represents the major cell population that differentiates into osteoclasts once recruited into arthritic joints (5). Since there were significantly fewer osteoclasts in the knee joints of mice treated with antibiotics and aIL-15ab than in controls, we assessed whether the therapy had an impact on these osteoclast precursors in the draining lymph nodes of the knee. There was an increased proportion of inflammatory Ly6C^high^ monocytes in the draining lymph nodes of mice treated with antibiotics and aIL-15ab compared to control mice (Fig. 3B). At the same time, there were no major differences in the proportions of other cell subsets in the draining lymph nodes (see Fig. S4 in the supplemental material). Lastly, no differences were obtained between the groups with respect to the proportions of CD11b^+^ cells, neutrophils, or inflammatory monocytes or the absolute cell numbers in the bone marrow (see Fig. S5 in the supplemental material). IL-15 has been shown to be important for monocyte recruitment to inflammatory sites since IL-15 stimulates monocytes to produce MCP-1, which leads to monocyte recruitment to inflamed areas (11). Indeed, we found that the serum levels of MCP-1 were reduced in mice treated with antibiotics and aIL-15ab compared to control mice (Fig. 3C).

Taken together, the combination therapy of antibiotics and aIL-15ab leads to an increased proportion of inflammatory monocytes in the draining lymph nodes and reduced serum levels of MCP-1 compared to control treated mice. This suggests that these cells are not recruited into the inflamed joint to subsequently differentiate to bone-eroding osteoclasts, explaining the reduced numbers of osteoclasts in the joint and diminished bone erosions during S. aureus-induced arthritis.

## DISCUSSION

In this study, we have shown that the addition of aIL-15ab to antibiotics as a treatment of S. aureus-induced arthritis reduced synovitis and bone erosions, as well as the number of osteoclasts in the joints, without compromising the host's ability to clear the infection. Thus, although IL-15 is a cytokine that is important for innate immune responses that are protective in the case of staphylococcal infections, aIL-15ab ought to be a safe addition to antibiotics.

S. aureus-induced arthritis is characterized by a very severe and rapid joint destruction that often leads to disabling joint damage despite antibiotics (1). In systemic infection with S. aureus, bacterial elimination and joint destruction are mediated by the activation of cells in the immune system, with a substantial release of proinflammatory proteins essential for elimination of the invading pathogen. The same immune response can, on the other hand, be harmful to the host. In fact, eliminating the bacteria is not sufficient to prevent the joint damage and, moreover, antibiotic-killed bacteria can induce and maintain joint inflammation (31). Therefore, it is tempting to seek therapeutic approaches that could tip the balance to a more favorable outcome where the destructive processes could be avoided without negatively influencing the protective host response in clearing the infection. Many agents have been investigated to obtain this goal. Pretreatment with an IL-1R antagonist aggravated S. aureus-induced arthritis with a more pronounced bone destruction and synovitis and a higher mortality than controls (26). The combination of antibiotics and a TNF-α inhibitor attenuated the postinfectious sequelae in S. aureus-induced arthritis when administered 3 days after the initiation of the infection (28). However, pretreatment with a TNF-α inhibitor worsened the outcome of S. aureus-induced arthritis compared to controls, with a 30-fold increase in bacterial load in the kidneys and a more pronounced weight loss (27). Therefore, it is desirable to pursue other immunomodulatory agents that have a lower potential to interfere with the protective immune response. Based on our results, IL-15 is a promising candidate. Indeed, in the case of sepsis, IL-15 inhibition is beneficial in both polymicrobial and endotoxin-induced sepsis (22, 23), although in the case of Gram-positive S. aureus-induced sepsis, IL-15 knockout mice have survival rates similar to those of wild-type mice (19). Further along these lines, Su et al. recently advocated for an IL-15 antagonist as a therapeutic agent in Stevens-Johnson syndrome and toxic epidermal necrolysis, which are life-threatening immune-related adverse drug reactions mediated by cytotoxic T lymphocytes and NK cells where IL-15 has been shown to be elevated and correlated with the progression and fatality of these severe hypersensitivity reactions (32).

Inflammatory processes, both acute and chronic, are often accompanied by bone loss, and inflammation itself is a trigger for the formation of osteoclasts, bone erosions, and in some cases, systemic bone loss (3). IL-15 plays a role in osteoclastogenesis which seems to be overall pro-osteoclastogenic (12–14), but the results are somewhat contradictory. Although IL-15-activated NK cells from the synovial fluid of rheumatoid arthritis patients efficiently induced osteoclast formation from autologous monocytes (15), IL-15-activated NK cells from healthy donors triggered osteoclast apoptosis, thus displaying an anti-osteoclastogenic effect (33). We and others have also observed an anti-osteoclastogenic effect of IL-15 *in vitro* (28, 34; B. Bergmann, unpublished data). These contrasting findings could mean that IL-15 displays paradoxical roles in osteoclastogenesis depending on the physiology, the presence of inflammation, and the inflammatory cytokine profile, and therefore the precise effects of IL-15 on osteoclastogenesis remain to be fully elucidated.

We have both previously (19) and in the present study shown that the inhibition of IL-15 reduces the number of osteoclasts in the joint and the subsequent bone destruction during S. aureus-induced arthritis. Ly6C^high^ monocytes comprise precursor cells that differentiate to osteoclasts once recruited into the inflamed joint (5). Mice receiving antibiotics and aIL-15ab had higher proportions of Ly6C^high^ inflammatory monocytes in their draining lymph nodes and fewer osteoclasts in their arthritic joints than controls. IL-15 has been shown to be important for monocyte recruitment to inflammatory sites as IL-15 stimulates monocytes to produce MCP-1, which leads to monocyte recruitment to inflamed areas (11). Since the serum levels of MCP-1 were reduced in mice receiving the combination therapy, we believe that the neutralization of IL-15 leads to an impaired production of MCP-1, followed by reduced recruitment of inflammatory monocytes to the joint and thus reduced differentiation into bone-eroding osteoclasts within the joint, resulting in reduced bone erosions.

Synovitis was reduced in mice receiving the combination therapy, and the proportion of granulocytes in the synovium was lower. IL-15 influences neutrophil recruitment to inflammatory sites by modulating chemokine activity and inhibits neutrophil apoptosis (10, 11). It is therefore not surprising that IL-15 inhibition reduces the proportion of granulocytes in the synovium of the arthritic joint. The question is whether the combination therapy leads to fewer osteoclasts in the joint via an inhibitory effect on synovial inflammation, consequently leading to a reduced osteoclast differentiation, or whether the effect is more directly targeted to the bone erosive process. There were no differences between the treatment groups in synovial mRNA levels of RANK, RANKL, or OPG at day 12 postinfection. This suggests that IL-15 does not interfere with this molecular triad at this time point. Importantly, the reduction of synovitis in mice treated with aIL-15ab and antibiotics was not accompanied by a significant reduction in cartilage destruction. Cartilage degradation is mediated by the inflammation-induced secretion of matrix-degrading enzymes, such as metalloproteinases (6). Notably, cartilage and bone destructive processes occur through distinct mechanisms, since in the absence of osteoclasts during arthritic conditions, the bone erosive process is arrested without affecting cartilage degradation or synovial inflammation (35, 36). This could indicate that the effect of IL-15 inhibition on bone erosion is directly mediated through reduced osteoclastogenesis in the joint or the reduced recruitment of osteoclast precursors to the joint.

The antibiotic regime was chosen in order to mimic the clinical setting, where patients are started on i.v. antibiotics at diagnosis and continued until they are stabilized and later changed to oral antibiotics. However, one limitation of the study design is that the oral antibiotic consumption of each individual mouse was not tracked.

The irreversible nature of the joint damage in S. aureus-induced arthritis, despite antibiotics, spurs a need for new therapeutic procedures. The inhibition of IL-15 and the use of antibiotics synergize in reducing synovitis and joint destruction in cases of S. aureus-induced septic arthritis and, despite the fact that the combination therapy does not significantly influence cartilage destruction, this approach comprises a possible novel and safe treatment option.

## MATERIALS AND METHODS

### Mice.

Eight-week-old female wild-type C57BL/6 mice were obtained from Scanbur (Sollentuna, Sweden). Previous studies have shown that male and female mice respond similarly to S. aureus-induced arthritis (19). Mice were maintained under standard conditions of temperature and light and were fed laboratory chow and water *ad libitum* at the animal facility at the Department of Rheumatology and Inflammation Research at the University of Gothenburg, Gothenburg, Sweden. The local Animal Research Ethics Committee, in accordance with national animal welfare legislation, approved all of the animal procedures (121213 353-2012 and 110928 378-2011).

### Mouse model of systemic S. aureus-induced arthritis.

The toxic shock syndrome toxin 1 (TSST-1)-producing LS-1 strain of S. aureus was used for infection (37). Female wild-type C57BL/6 mice were inoculated i.v. in the tail vein with 0.8 × 10^8^ CFU S. aureus LS-1/mouse in a total volume of 200 μl of phosphate-buffered saline (PBS). To determine the number of bacteria injected, viable counts were performed. Each mouse was weighed and examined daily for assessment of its overall condition, for clinical signs of arthritis, and for signs of systemic infection. During the course of the infection, the mice were graded blindly for clinical arthritis as previously described (19).

In cases of severe systemic infection, when a mouse was judged too ill to survive another 24 h, it was culled and defined as dead due to sepsis. Blood, bone marrow, kidneys, limbs, draining lymph nodes of the knee, and synovial tissue from knee joints were obtained at 12 days postinfection for further analysis.

### Treatment with antibiotics and anti-IL-15 antibodies.

IL-15 neutralizing antibodies were kindly provided by Amgen. Treatment was started on day 3 after bacterial inoculation and consisted of anti-IL-15 antibodies (aIL-15ab; Amgen, Inc., Thousand Oaks, CA) with antibiotics for the combination therapy group and isotype control antibodies (IgG2a; Amgen) with antibiotics for the control group. This experiment was repeated three times, with 10 mice in each of the two groups. In an additional experiment, there were four treatment groups (*n* = 10 mice in each group) which are described in Table 1. The antibodies (25 μg/mouse) were injected intraperitoneally at days 3, 6, and 10 after bacterial inoculation. Intraperitoneal injections of cloxacillin (7.5 mg/mouse) were given twice daily from day 3 and stopped at day 6, when flucloxacillin was added to the drinking water (70 mg/kg). The rationale for starting treatment at day 3 is to simulate the treatment delay that is present in the clinical setting of septic arthritis. We also chose the antibiotic regime in order to mimic the clinical setting, wherein patients are started on i.v. antibiotics at diagnosis, and this treatment continues until the patients are stabilized, with a subsequent change to oral antibiotics. The peroral antibiotic mixture was changed every third day, and the treatment groups were mixed to avoid cage effect, i.e., that the cage microenvironment could influence the outcome irrespective of genotype (38).

**TABLE 1:** Study design^*a*^

Treatment combination	Treatment details			
	Group 1	Group 2	Group 3	Group 4
Antibody or isotype control	Isotype control antibody, i.p.	aIL-15ab, i.p.	Isotype control antibody, i.p.	aIL-15ab, i.p.
Antibiotics or control fluid	PBS control, i.p., days 3 to 6	PBS control, i.p., days 3 to 6	Cloxacillin, i.p. days 3 to 6, followed by oral flucloxacillin in drinking water	Cloxacillin, i.p., days 3 to 6, followed by oral flucloxacillin in drinking water

a i.p., intraperitoneal.

### Bacterial clearance.

S. aureus homes to joints and kidneys and persists there, and the bacterial load in the kidneys is therefore an indirect measurement of bacterial clearance. Kidneys were aseptically dissected, kept on ice, homogenized, serially diluted in PBS, and spread on blood agar plates. After 24 h of incubation at 37°C, the number of CFU per kidney pair was determined.

### Histopathology of inflamed joints.

The degree of cartilage destruction was assessed using Safranin O (the staining intensity of Safranin O is directly proportional to the proteoglycan content in the cartilage) (39). Bone erosions were detected using Weigert's hematoxylin Van Gieson procedure of differential staining of collagen and other connective tissue (40). Joints were fixated, decalcificated, and paraffin embedded. Tissue sections from fore- and hindpaws were cut, deparaffinized, and stained with hematoxylin-eosin (Histolab Products AB, Gothenburg, Sweden) or Weigert's iron hematoxylin (Histolab Products AB, Gothenburg, Sweden) prior to Van Gieson staining or Safranin O (Sigma-Aldrich AB, Stockholm, Sweden) staining with Fast Green counterstaining (40). All slides were coded, and two blinded observers evaluated each slide. The specimens were evaluated with regard to inflammatory cell accumulation in synovial tissue (synovitis), cartilage destruction, and bone erosions. The degree of synovitis and destruction yielded a score from 0 to 3 in every joint concerning finger/toes, wrists/ankles, elbows, and knee. Occasionally, one paw was embedded in a way that made it impossible to evaluate, and therefore the total score/mouse is divided by the number of evaluated joints.

### Immunohistochemical osteoclast staining.

The number of osteoclasts in femur and tibia epiphyses was determined by staining for cathepsin K, a proteolytic enzyme predominantly expressed in osteoclasts, as previously described (41). All slides were coded, and the number of cathepsin K-stained, multinucleated osteoclasts per 0.1 mm^2^ was counted in the epiphysis.

### Gene expression analysis.

Synovial tissue was obtained from the knee joints, and RNA was isolated using an RNeasy minikit (Qiagen) according to the manufacturer's instructions. The RNA quality was analyzed using an Experion Bioanalyzer on a Experion RNA StdSens chip (Bio-Rad Laboratories, Inc., Hercules, CA) prior to cDNA synthesis with a high-capacity cDNA reverse transcription kit (Applied Biosystems). The expression of *Rank*, *Rankl*, and *Opg* genes was measured with GAPDH (glyceraldehyde-3-phosphate dehydrogenase) as a reference gene. All reactions were amplified using TaqMan Gene expression PCR master mix (Applied Biosystems) and analyzed on a Viia7 system (Applied Biosystems).

### Flow cytometry.

Single cell suspensions were prepared from spleens, draining lymph nodes of the knee, bone marrow, and synovia and preincubated with Fc-block (BD Biosciences). The antibodies used are summarized in Table S1 in the supplemental material. Analyses were performed on a FACSCanto II, equipped with FACSDiva software (BD Bioscience), and with the FlowJo software (Tree Star, Inc.). The gating strategies were based on a fluorochrome minus one setting, when needed. Briefly, inflammatory monocytes were defined as CD11b^+^ Lineage^−^ (cells gated out using a B220, CD4, CD8, and NK1.1 fluorescein isothiocyanate [FITC] dump channel), Ly6C^high^, and Ly6G^−^ (42).

### ELISA.

Blood was collected at various time points after S. aureus inoculation, and serum was stored at −20°C for further analysis. The serum was analyzed for MCP-1 by using a DuoSet enzyme-linked immunosorbent assay (ELISA) development kit (mouse CCL2/JE/MCP-1 [DY479-05]; R&D Systems, Abingdon, UK) according to the manufacturer's recommendations. The assay was run on a Spectra Max 340PC (Molecular Devices, Sunnyvale, CA), and analysis was performed using SoftMax Pro 5.2 software (Molecular Devices).

### pQCT and assessment of bone mineral density.

Femoral bones were fixed in formalin, subsequently placed in 70% ethanol, and then subjected to peripheral quantitative computed tomography (pQCT) scanning with Stratec pQCT XCT Research M (software v5.4 B; Norland, Fort Atkinson, WI) at a resolution of 70 μm. The trabecular bone mineral density (BMD) was determined with a metaphyseal scan, performed at a distance from the growth plate corresponding to 3% of the length of the femur. The inner 45% of the area was defined as the trabecular bone compartment.

### Statistical analysis.

Statistical analyses were performed using Prism (GraphPad Software). Comparisons between groups were performed using a Mann-Whitney U test. Kaplan-Meier survival plots were prepared, and the log-rank test was used for comparisons between the survival curves. *P* < 0.05 was considered statistically significant.

## Supplementary Material

Supplemental material
