# Supplementary material for: Antibiotics with Interleukin-15 Inhibition Reduce Joint Inflammation and Bone Erosions but Not Cartilage Destruction in Staphylococcus aureus-Induced Arthritis
Source: Infect Immun. 2018 Apr 23;86(5):e00960-17. doi: 10.1128/IAI.00960-17 (PMC5913847; doi:10.1128/IAI.00960-17)
Supplement: Supplemental material [file supp_86_5_e00960-17__index.html]

Supplemental material 

# Antibiotics with Interleukin-15 Inhibition Reduce Joint Inflammation and Bone Erosions but Not Cartilage Destruction in Staphylococcus aureus-Induced Arthritis

## Supplemental material

- Supplemental file 1 -

  Fig. S1. The effects of anti-IL-15 antibodies (aIL-15ab) and antibiotic treatments on the clinical course of *Staphylococcus aureus*-induced arthritis and joint damage.

  PDF, 320K
- Supplemental file 2 -

  Fig. S2. Effects on the combination treatment of aIL-15ab and antibiotics on cortical density and thickness and the mRNA expression levels of *Rank*, *Rankl*, and *Opg* in the synovium 12 days after the initiation of *Staphylococcus aureus*-induced arthritis.

  PDF, 94K
- Supplemental file 3 -

  Fig. S3. Effects of the combination treatment of aIL-15ab and antibiotics on cell populations in the synovium during *Staphylococcus aureus*-induced arthritis.

  PDF, 92K
- Supplemental file 4 -

  Fig. S4. Effects on the combination treatment of aIL-15ab and antibiotics on cell populations in the draining lymph nodes of the knee during *Staphylococcus aureus*-induced arthritis.

  PDF, 134K
- Supplemental file 5 -

  Fig. S5. Effects on the combination treatment of aIL-15ab and antibiotics on cell populations in the bone marrow during *Staphylococcus aureus*-induced arthritis.

  PDF, 84K
- Supplemental file 6 -

  Table S1. Antibodies used for flow cytometry.

  PDF, 18K
- Supplemental file 7 -

  Fig. S1 to S5 legends.

  PDF, 101K
