# Supplementary figures and images for: Antibiotics with Interleukin-15 Inhibition Reduce Joint Inflammation and Bone Erosions but Not Cartilage Destruction in Staphylococcus aureus-Induced Arthritis
Source: Infect Immun. 2018 Apr 23;86(5):e00960-17. doi: 10.1128/IAI.00960-17 (PMC5913847; doi:10.1128/IAI.00960-17)

# Supplemental Figure 1

▲ Isotype + PBS      ▲····· aL-15ab + PBS  
 ○ Antibiotics + Isotype      ●····· aL-15ab + antibiotics

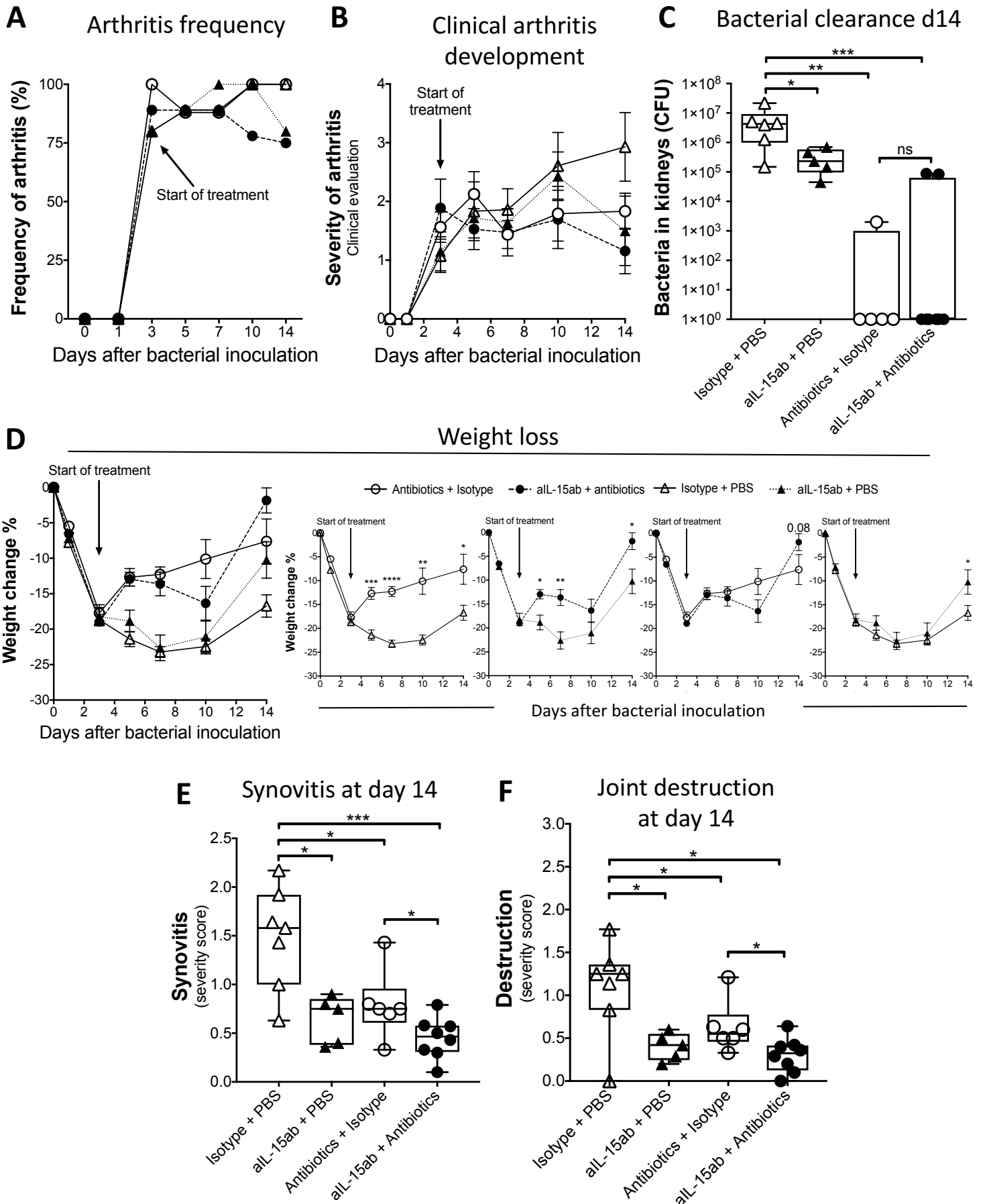

Supplement: Supplemental material [file IAI.00960-17_zii999092382s1.pdf]

## Supplemental Figure 2

○ A+Ctrl ab

● A+aIL-15ab

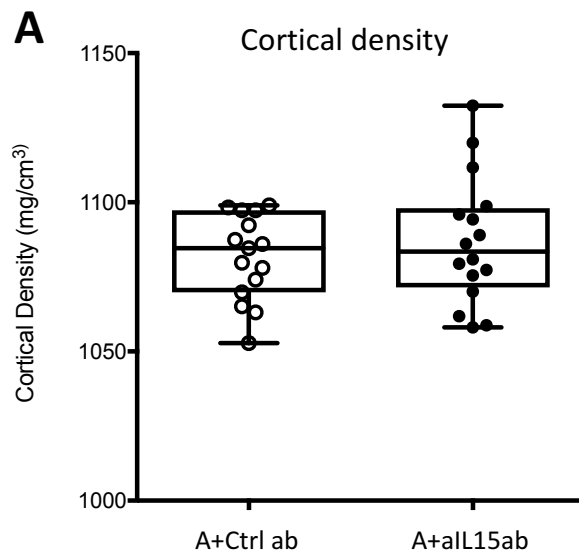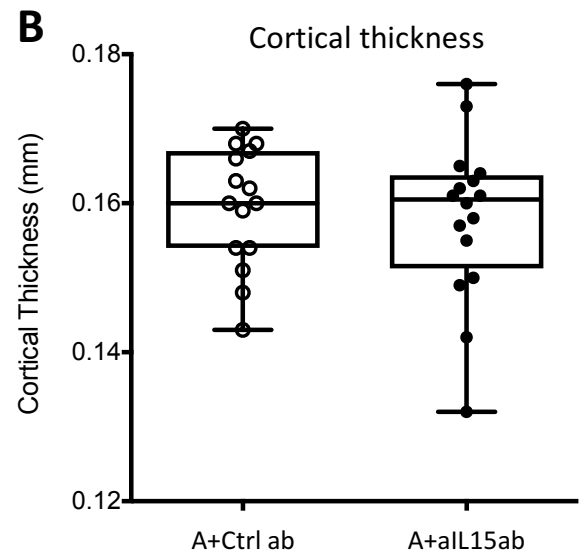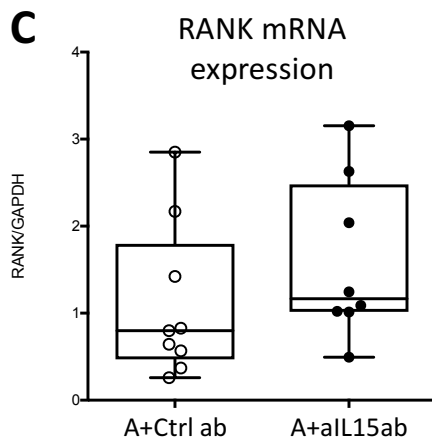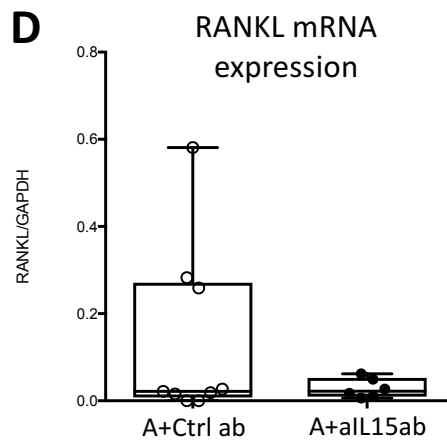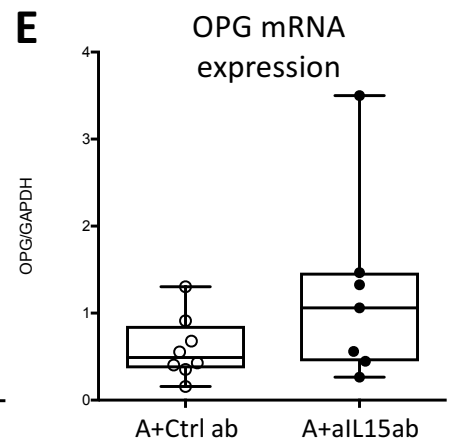

Supplement: Supplemental material [file IAI.00960-17_zii999092382s2.pdf]

# Supplemental Figure 3

Synovial membrane

○ A+Ctrl ab

● A+allL-15ab

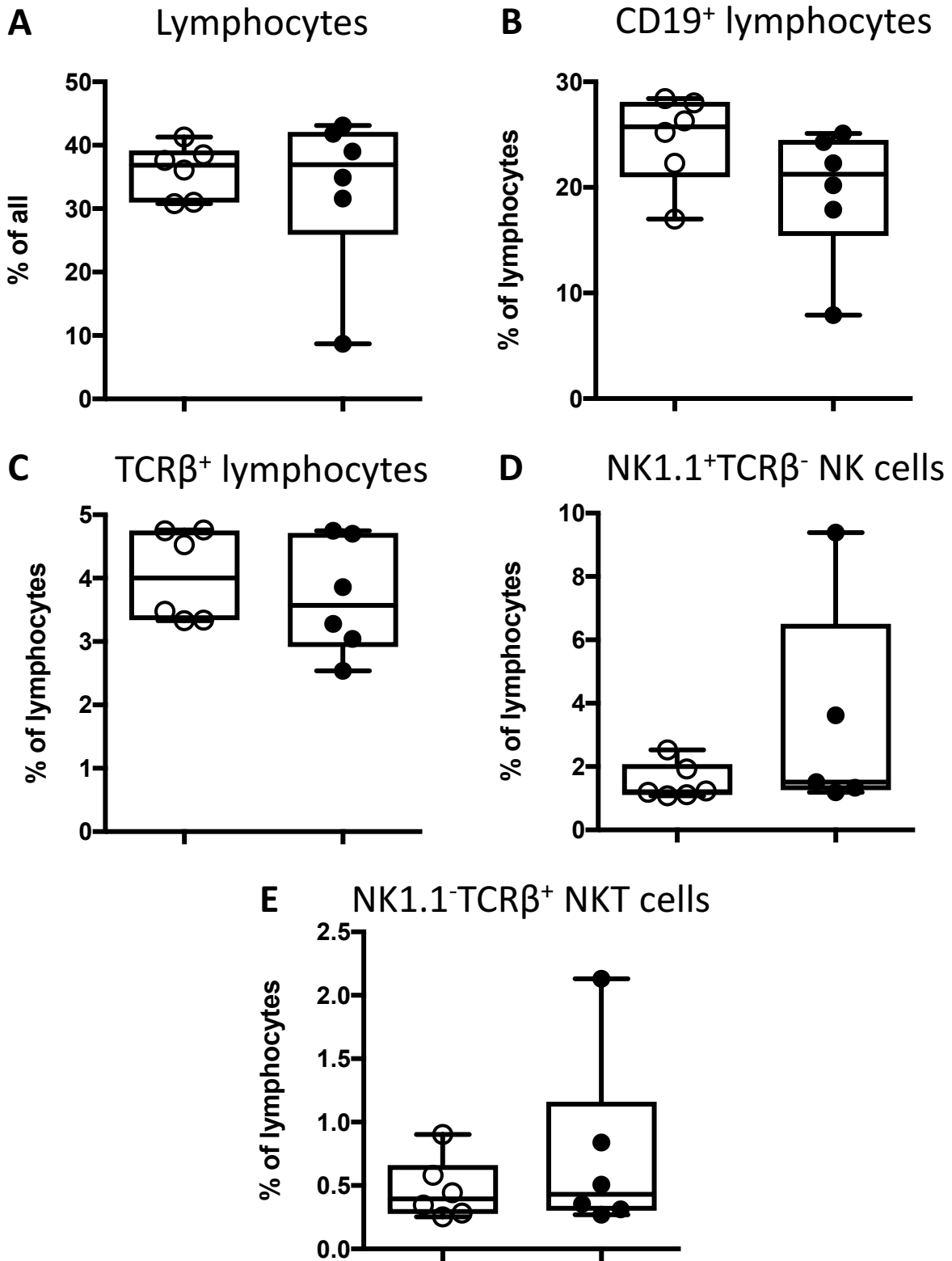

Supplement: Supplemental material [file IAI.00960-17_zii999092382s3.pdf]
