# Supplementary material for: Antibiotics with Interleukin-15 Inhibition Reduce Joint Inflammation and Bone Erosions but Not Cartilage Destruction in Staphylococcus aureus-Induced Arthritis
Source: Infect Immun. 2018 Apr 23;86(5):e00960-17. doi: 10.1128/IAI.00960-17 (PMC5913847; doi:10.1128/IAI.00960-17)

Supplemental Figure 4

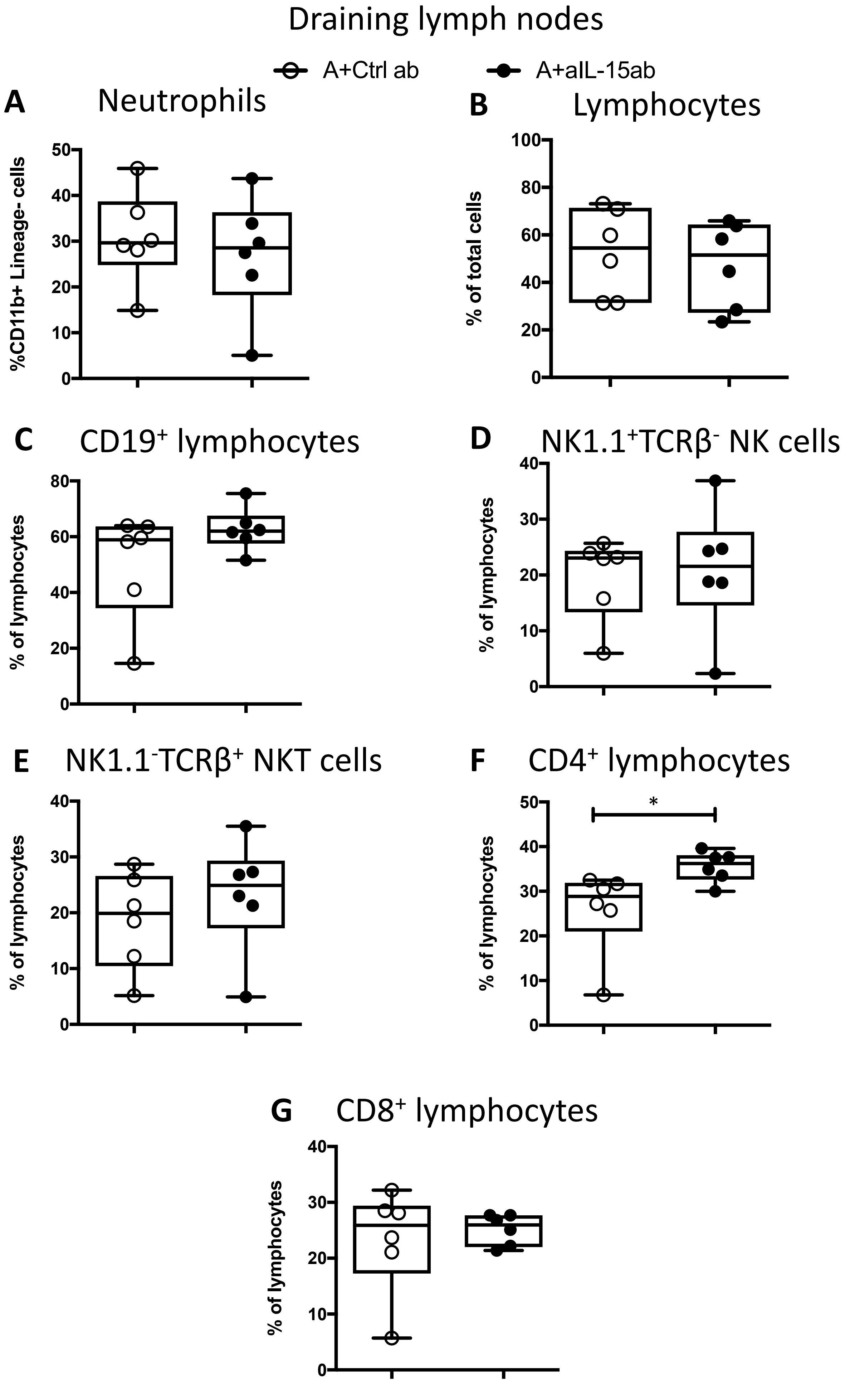

Supplement: Supplemental material [file IAI.00960-17_zii999092382s4.pdf]
