# Supplementary material for: Antibiotics with Interleukin-15 Inhibition Reduce Joint Inflammation and Bone Erosions but Not Cartilage Destruction in Staphylococcus aureus-Induced Arthritis
Source: Infect Immun. 2018 Apr 23;86(5):e00960-17. doi: 10.1128/IAI.00960-17 (PMC5913847; doi:10.1128/IAI.00960-17)
Supplement: Supplemental material [file IAI.00960-17_zii999092382s6.pdf]

Supplemental Table 1.

Antibodies used for flow cytometry

Mouse Antigen	Conjugate	Clone	Source
TCRb	APC	H57-597	eBioscience
TCRb	PE	H57-597	eBioscience
Streptavidin	FITC		eBioscience
CD8a	PE	53-6.7	eBioscience
CD115	APC	AFS98	eBioscience
CD11c	APC	N418	eBioscience
CD19	PE-Cy7	1D3	eBioscience
Ly6C	APC-Cy7	AL-21	BD Biosciences
CD11b	v450	M1/70	BD Biosciences
NK1.1	biotin	PK136	BD Biosciences
CD4	v450	RM4-5	BD Biosciences
CD11c	PECy7	HL3	BD Biosciences
Ly6G	PE	IA8	BD Biosciences
B220	FITC	RA3-6B2	BD Pharmingen
CD8a	FITC	53-6.7	BD Pharmingen
CD4	FITC	H129.19	BD Pharmingen
