# Supplementary material for: Antibiotics with Interleukin-15 Inhibition Reduce Joint Inflammation and Bone Erosions but Not Cartilage Destruction in Staphylococcus aureus-Induced Arthritis
Source: Infect Immun. 2018 Apr 23;86(5):e00960-17. doi: 10.1128/IAI.00960-17 (PMC5913847; doi:10.1128/IAI.00960-17)
Supplement: Supplemental material [file IAI.00960-17_zii999092382s7.pdf]

Supplemental Figure Legends

Supplemental Figure 1. The effects of anti-IL-15 antibodies (aIL-15ab) and antibiotic treatments on the clinical course of *Staphylococcus aureus*-induced arthritis and joint damage.

Mice were intravenously inoculated with *S. aureus* LS-1 and treated 3 days later with 1) aIL-15ab with PBS control injection for antibiotic fluid from d3-d6; 2) Isotype control antibody with PBS control injection for antibiotic fluid from d3-d6; 3) Isotype control antibody with antibiotics; 4) aIL-15ab with antibiotics (n = 10 mice/treatment group). The antibodies were injected intraperitoneally at days 3, 6, and 10 postinfection. The frequency (A) and severity of arthritis (B) was evaluated during the course of the infection. (C) Bacterial persistence in the kidneys at 14 days postinfection. (D) Weight change as percentage of initial weight. The severity of joint inflammation, or synovitis, and joint destruction was evaluated by making a histological scoring of synovitis (F) and joint destruction (G). In panels A, B and D, bars show the mean ± standard error of the mean (SEM). In panels C, F and G, data is shown as median, whiskers = min to max. Statistical differences were calculated using Mann-Whitney U test. Kaplan-Meier survival plots were prepared and the log-rank test was used for comparison between the two survival curves. *$p < 0.05$; **$p<0.01$; ***$p<0.001$. aIL-15ab, anti-IL-15 antibodies; CFU, colony-forming units; ns, not significant. Groups 1 and 2 have previously been published (Henningsson L, Jirholt P, Bogestal YR, Eneljung T, Adiels M, Lindholm C, McInnes I, Bulfone-Paus S, Lerner UH, Gjertsson I, Interleukin 15 mediates joint destruction in Staphylococcus aureus arthritis, J Infect Dis, 2012, 206:687-696, by permission of Oxford University Press).

Supplemental Figure 2. Effects on the combination treatment of aIL-15ab and antibiotics on cortical density and thickness, and the mRNA expression levels of *Rank, Rankl, and*

***Opg* in the synovium 12 days after the initiation of *Staphylococcus aureus*-induced arthritis.**

Cortical density (A) and thickness (B) of the femur measured with pQCT. Relative mRNA expression levels of the molecular triad of *Rank* (C), *Rankl* (D), and *Opg* (E) in synovium of the knee joints 12 days after initiation of *S. aureus*-induced arthritis. Horizontal bars show median values, whiskers = min to max. Statistical differences were calculated using Mann-Whitney U test.

Supplemental Figure 3. Effects of the combination treatment of aIL-15ab and antibiotics on cell populations in the synovium during *Staphylococcus aureus*-induced arthritis.

The proportion of lymphocytes (A), $CD19^+$ B lymphocytes (B), $TCR\beta^+$ T lymphocytes (C), NK cells (D), and NKT cells (E) in the synovium of the knee joints 12 days after initiation of *S. aureus*-induced arthritis. Horizontal bars show median values, whiskers = min to max. Statistical differences were calculated using Mann-Whitney U test.

Supplemental Figure 4. Effects on the combination treatment of aIL-15ab and antibiotics on cell populations in the draining lymph nodes of the knee during *Staphylococcus aureus*-induced arthritis.

The proportions of neutrophils (A), lymphocytes (B), $CD19^+$ B cells (C), NK cells (D), NKT cells (E), $CD4^+$ T cells (F), and $CD8^+$ T cells (G) in the draining lymph nodes of the knee at day 12 after initiation of *S. aureus*-induced arthritis. Horizontal bars show median values, whiskers = min to max. Statistical differences were calculated using Mann-Whitney U test. *$p<0.05$, compared with control group.

Supplemental Figure 5. Effects on the combination treatment of aIL-15ab and antibiotics

on cell populations in the bone marrow during *Staphylococcus aureus*-induced arthritis. The proportions of CD11b^{+} cells (A), neutrophils (B), Ly6C^{high} inflammatory monocytes (C), and the absolute cell number (D) in the bone marrow at day 12 after initiation of *S. aureus*-induced arthritis. Horizontal bars show median values, whiskers = min to max. Statistical differences were calculated using Mann-Whitney U test.
